# A64 THE DIETARY FIBRE RHAMNOGALACTURONAN PROMOTES INTESTINAL EPITHELIAL CELL MIGRATION THROUGH THE NF-κB SIGNALING PATHWAY

**DOI:** 10.1093/jcag/gwab049.063

**Published:** 2022-02-21

**Authors:** C H Baggio, J Shang, A Nascimento, T Cipriani, W MacNaughton

**Affiliations:** 1 Physiology and Pharmacology, University of Calgary, Calgary, AB, Canada; 2 Federal University of Acre, Cruzeiro do Sul, Acre, Brazil; 3 Universidade Federal do Parana, Curitiba, PR, Brazil; 4 Univ Calgary, Calgary, AB, Canada

## Abstract

**Background:**

Damaged intestinal epithelial barrier is characteristic of inflammatory bowel diseases (IBD) and mucosal healing is the primary goal for IBD treatment. We previously showed the direct beneficial effects of rhamnogalacturonan (RGal), a polysaccharide isolated from the plant *Acmella oleracea*, on intestinal epithelial barrier function with participation of TLR4 and PKC activation. We also observed that RGal accelerates wound healing in human colonic epithelial Caco-2 cells. RNAseq data and pathway analysis have indicated the involvement of the canonical nuclear factor kB (NF-kB) signaling pathway.

**Aims:**

We hypothesize that RGal increases intestinal epithelial wound healing through NF-kB signaling pathway.

**Methods:**

Caco-2 cells monolayers were scratched and treated with vehicle (media or 0.5% DMSO in media) or RGal (1000 mg/ml) for 48 h. Wound healing was assessed using the IncuCyte live cell imaging system. Proliferation and apoptosis of cells were evaluated using EdU and TUNEL assays, respectively. Inhibitors were added at the same time (transcription inhibitor Actinomycin D, 5 mg/ml) or 1 h (NF-kB inhibitors Bay 11–7082 and JSH-23, 10 and 20 mM, respectively, or COX-2 inhibitor NS-398, 20 mM) before RGal treatment. Unwounded Caco-2 monolayers treated with RGal (1000 mg/ml) were collected for Western blotting for COX-2 protein.

**Results:**

In the wound healing assay, RGal at a concentration of 1000 µg/ml enhanced wound healing by 12.5% at 48 h compared to control group, under 10% serum conditions. Neither proliferation nor apoptosis were involved in the RGal effect on wound healing, suggesting the response was due solely to cell migration. Actinomycin D (5 mg/ml), Bay 11–7082 (10 mM) or JSH-23 (20 mM) treatment significantly reversed the effect of RGal on wound healing, showing that the response was transcriptionally dependent and involved NF-kB signaling. Treatment of cells with NS-398 (20 mM) also reversed the effect of RGal on wound healing. COX-2 protein expression was significantly increased at 6 and 12 h after RGal addition to Caco-2 monolayers.

**Conclusions:**

These data suggest that the plant-based polysaccharide RGal increases intestinal epithelial cell wound healing by increasing cell migration. The RGal effect is dependent on the activation of the transcription factor NF-kB and downstream COX-2 protein expression and activity. Our findings show a novel mechanism of action of RGal in wound healing that could help in the resolution of intestinal inflammation and mucosal healing.

**Funding Agencies:**

NSERC

